# Linking Social Cognition, Parvalbumin Interneurons, and Oxytocin in Alzheimer’s Disease: An Update

**DOI:** 10.3233/JAD-230333

**Published:** 2023-11-21

**Authors:** Daniela Černotová, Karolína Hrůzová, David Levčík, Jan Svoboda, Aleš Stuchlík

**Affiliations:** a Laboratory of Neurophysiology of Memory, Institute of Physiology of the Czech Academy of Sciences, Prague, Czech Republic; b Third Faculty of Medicine, Charles University, Prague, Czech Republic

**Keywords:** Alzheimer’s disease, animal models, dementia, hippocampus, oxytocin, parvalbumin interneurons, social cognition, social memory

## Abstract

Finding a cure for Alzheimer’s disease (AD) has been notoriously challenging for many decades. Therefore, the current focus is mainly on prevention, timely intervention, and slowing the progression in the earliest stages. A better understanding of underlying mechanisms at the beginning of the disease could aid in early diagnosis and intervention, including alleviating symptoms or slowing down the disease progression. Changes in social cognition and progressive parvalbumin (PV) interneuron dysfunction are among the earliest observable effects of AD. Various AD rodent models mimic these early alterations, but only a narrow field of study has considered their mutual relationship. In this review, we discuss current knowledge about PV interneuron dysfunction in AD and emphasize their importance in social cognition and memory. Next, we propose oxytocin (OT) as a potent modulator of PV interneurons and as a promising treatment for managing some of the early symptoms. We further discuss the supporting evidence on its beneficial effects on AD-related pathology. Clinical trials have employed the use of OT in various neuropsychiatric diseases with promising results, but little is known about its prospective impacts on AD. On the other hand, the modulatory effects of OT in specific structures and local circuits need to be clarified in future studies. This review highlights the connection between PV interneurons and social cognition impairment in the early stages of AD and considers OT as a promising therapeutic agent for addressing these early deficits.

## INTRODUCTION

Alzheimer’s disease (AD) is a neurodegenerative disease with an enormous socioeconomic burden. Almost 60 million people worldwide suffer from this disease, and the numbers are expected to triple by 2050 [1]. The main histopathological hallmarks of AD include the extracellular deposits of insoluble amyloid-β protein (amyloid-β plaques) and intracellular aggregates of hyperphosphorylated tau protein, accompanied by neuronal loss and gradual atrophy of the brain [2]. Progressive memory loss is often referred to as the most prominent symptom. However, besides the gradually degrading episodic memory, patients diagnosed with AD often experience affective and depressive symptoms, including mood swings, apathy, anxiety, and social withdrawal [3–5].

In this review, we focus on deficits in social cognition in early AD and relate them to the progressive decline of parvalbumin (PV) interneurons, also referred to as fast-spiking interneurons. To this date, few studies have addressed the relationship between PV interneurons and deficits in the social cognitive domain. We focus the review on rodent AD models, which form an indispensable part of current research. In the last section, we discuss oxytocin (OT) as a potent modulator of both PV interneurons and social memory, with additional beneficial effects against AD-related pathology.

### Early stage of AD and social behavior

Temporal lobes have long been considered the first morphologically and functionally affected areas, primarily the entorhinal cortex, amygdala [6–8], and the hippocampus [9–10]. These are the first structures affected by the extracellular amyloid-β plaque accumulation, the main pathological hallmark of AD [11]. Moreover, their volume is already significantly reduced with the onset of the first cognitive symptoms [8]. Besides their roles in cognition and memory, they are essential for emotional processing and social behaviors [12, 13].

According to Jost and Grossberg, social withdrawal is one of the earliest noticeable symptoms, occurring up to almost three years before the diagnosis in 40% of AD patients [3]. In addition, recent cohort studies confirmed that social isolation, such as living alone or not participating in social activities, dramatically increases the risk of developing dementia and even speeds its progression [14–16]. This forms a vicious cycle of social behavior dysfunction with the disease. Dissociating early symptoms from the causes of the disease yet remains a question of future research.

### Animal models and early AD

Most current animal AD models are based on inserting one or more gene mutations associated with an early-onset form of AD into the rodent genome. These include mutations in amyloid-β precursor protein (APP) in hAPPJ20 [17], APP^NL - F^ [18], and Tg2576 mice [19], or combined APP and presenilin mutations in 5xFAD [20] and APP/PS1 mice [21] and TgF344-AD rats [22]. Such insertions in these models generally lead to progressive amyloidosis and age-dependent cognitive decline [18–22]. Models involving tauopathies do exist, but amyloid models vastly outnumber them. In any case, AD is a multifactorial disease, and it is impossible to completely mimic the entire pathology in rodents, regardless of the model used. It is necessary to carefully interpret results from animal studies, as they are mostly limited to modeling early-onset familial AD, which involves only 5–6% of dementias [23]. The research also comprises various types of animal models, strains, and animals themselves, including different ages when undergoing the experiments. Despite comparable pathological features, the overall manifestation and progression of the disease can differ. Nevertheless, many pathological and behavioral alterations overlap in these AD models, including those in social behavior.

Similar to human patients with AD, rodent models also often exhibit early deficits in the social domain, preceding dysfunction in spatial memory. Reduced sociability and social recognition were reported in various AD models, starting as soon as three months [24–28] in both mice and rats. A study directly comparing social and spatial behavior found that 6-month-old APP and APP/PS1 female mice showed reduced sociability but no impairment in spontaneous spatial alternation [29]. Similar social deficits were observed in another study, where the APP/PS1 mice of the same age were less interested in social encounters per se but the authors also reported a hyperactivity in tests for general exploratory activity [30]. Progressive worsening of spatial learning and reference memory then first appears around five to six months [31–34], although some authors reported memory deficits starting even later— around nine to ten months of age in mice [19, 35, 36] and seven to eight months in rats [37]. Nevertheless, considerable variability is observed when probing deficits related to spatial learning. This variability seems to be influenced by a number of factors, namely various experimental design, evaluated parameters or specific genetic background of AD models used. Altogether, early differences in the social domain across various rodent models appear to be more consistent.

When addressing the impact of social isolation on AD progression in mice, it was shown that three-month long isolation from their conspecifics significantly increases plaque deposits in the hippocampus and worsens memory performance [38–40]. Apparently, there is a strong interconnection between the lack of social interactions and the progression of the disease in both human patients and animal models. The early symptoms of genetic animal models more or less resemble that of humans. Yet, deviations related to emotional states and social cognition have not received adequate attention in research, although examining them in the context of the ongoing histopathological changes could significantly contribute to early intervention.

### PV interneurons and E/I balance in AD

Numerous brain pathologies are characterized by an imbalance between excitatory and inhibitory actions in brain microcircuits, and AD is no exception. The excitatory/inhibitory (E/I) balance refers to the constant ratio of excitatory and inhibitory currents on individual pyramidal neurons [41]. Maintaining this balance in homeostasis is critical for efficient neural coding in the brain [42]. The equilibrium, however, undergoes dynamic modulation through short-term plasticity, influencing the strength and timing of individual synaptic inputs and modulating the signal outputs in the hippocampal circuits [43, 44]. A precisely controlled E/I balance is also essential for the formation and control of neuronal oscillations [45, 46]. E/I balance is primarily maintained by PV interneurons that modulate and synchronize pyramidal neurons [47–49]. In the hippocampus, these interneurons contribute to the generation of theta (4–10 Hz) [48] and gamma (30–120 Hz) [49] oscillations, along with sharp-wave ripples (150–200 Hz) [50]. These oscillations are crucial for long-term memory formation and information processing [51, 52].

Dysfunction of PV interneurons in the hippocampus inevitably leads to alterations in theta and gamma oscillations, which in turn may disrupt spatial working and reference memory [53]. Furthermore, optogenetic inhibition of PV interneurons also inhibits sharp wave-ripples [50], which are crucial for consolidating hippocampus-dependent memories [54]. The loss of PV interneurons or the loss of their proper functioning is accompanied by network disturbance and cognitive dysfunction in various psychiatric diseases [55]. Although the AD pathology was studied primarily in terms of dysfunction of glutamatergic and cholinergic systems, there is growing evidence that GABAergic, i.e., inhibitory signaling is affected in the early stages of this disorder, leaving a negative impact on the E/I balance [27, 55–60].

Disrupted E/I balance or altered function of PV interneurons was reported in various AD models [61–64]. Furthermore, reduced inhibition is linked to hypersynchronous or epileptiform activity, which was observed in these models as well [65–68]. In fact, altered excitability of PV interneurons in AD occur long before memory deficits appear, especially in the hippocampus [69–70]. Some authors suggest that aberrations in PV interneuron functioning might precede the increasing amyloid-β plaque accumulation [71–72], which starts at approximately four months in APP/PS1 mice [73] and six months in APP/PS1 rats [22]. On the other hand, the presence of amyloid-β might further aggravate the disrupted GABAergic activity and contribute to reduced dendritic inhibition of pyramidal cells in the hippocampus [74]. Findings confirming the early disruption of PV interneurons in AD contribute to emerging efforts to map their role in social cognition.

### PV interneurons and memory

PV interneurons play an important role in various types of memory, including spatial, fear, and social memory. In the hippocampal area CA1, which is well known for processing spatial information, these interneurons are crucial for spatial working memory but do not seem to be involved in spatial reference memory [75, 76]. In addition, mice with NMDA receptor ablation on PV interneurons demonstrated impaired spatial working memory and both short- and long-term object recognition memory, but intact spatial reference learning [53]. Despite some debate regarding the role of PV interneurons in phase-dependent recurrent inhibition in the medial entorhinal cortex [77], it has been shown that PV interneurons are necessary for maintaining the grid cells’ activity in this area, which is crucial for spatial representation [78]. PV interneurons were also studied in the context of memory consolidation. Specifically, they were shown to be critical for fear memory consolidation. They serve as the cellular substrate for preserving fear memories and are essential for associated high-frequency oscillatory activity in the hippocampus [79]. These interneurons also promote network plasticity in hippocampal CA1 and, consequently, long-term fear memory formation [51]. Furthermore, successful fear memory consolidation relies on coherent communication between the hippocampus and the neocortex, which is mediated by PV interneurons [80].

Recently, it was shown that PV interneurons might be a core of social memory in various brain areas, specifically the hippocampal dorsal CA2 and its connected ventral CA1 regions [81–84]. The dorsal CA2 is enriched in PV interneurons compared to other hippocampal areas [85]. This area is critical for the encoding, consolidation, and recall processes related to social memories [81]. The “social novelty signal” arises from the activity of the supramammillary nucleus of the hypothalamus, which sends its projections to the CA2 [86, 87]. This pathway provides inhibitory tone by stimulating PV basket interneurons and synchronizes the firing of pyramidal neurons [87]. Contrary to dorsal CA2, ventral CA1 only stores social memory engrams [82]. Higher calcium activity of PV interneurons in ventral CA1 was observed when approaching novel mice, while hyperexcitability or inactivation of these interneurons impaired social discrimination [83]. Interfering with the activity of these interneurons in any direction thus affects the ability to identify familiar and stranger conspecifics.

Long-term depression is a type of plasticity unique to the hippocampal CA2 region [88]. This type of plasticity depends on maturation of PV interneurons during adolescence, mainly due to receptor tyrosine-protein kinase ErbB4 signaling and the formation of perineuronal nets around PV interneurons [84]. Inhibitory plasticity appears to be the core mechanism for social memory formation [84]. Indeed, Rey and colleagues confirmed this finding by showing that the disrupted function of PV interneurons and loss of perineuronal nets in CA2 led to social memory deficits in Tg2576 mice, a double mutant AD model [27]. Moreover, the authors reported lower numbers of PV interneurons and lower intensities of fluorescent anti-PV staining in these cells [27]. Interestingly, dysfunction of PV interneurons was not manifested in deficits in spatial memory, confirming the exclusivity of CA2 in social memory processing [27]. On the other hand, chronic but not acute silencing of PV interneurons in ventral CA1 affected the recognition of novel objects [83]. Thus, chronic manipulation of PV interneurons in ventral CA1 may impact both social and non-social aspects of memory.

Various neuromodulators are involved in social coding. Activation of delta-opioid and cannabinoid type 1 receptors induces long-term depression of inhibitory transmission in the CA2, which is required for social memory formation [88, 89]. Furthermore, oxytocinergic signalization modulates both excitatory pyramidal cells and PV interneurons in the CA2 [90]. Activation of hippocampal OT receptors (OTRs) is critical for discriminating between social memories [91] and maintaining long-term social recognition memory [92].

As mentioned previously, prolonged social isolation of either young or old mice with AD-related genes can exacerbate the progression of the disease [38–40]. Deng et al. reported that social isolation also reduces the PV interneuron number in the ventral hippocampus [83]. Thus, social isolation could be another factor that negatively affects interneurons, making them vulnerable to pathological events. Altogether, the activity of PV interneurons is not only crucial for social memory but also plays significant roles in various aspects of spatial and declarative memory processes. Gradual impairment of these interneurons will undoubtedly manifest across many cognitive domains.

### PV interneuron dysfunction in the early stage

In the earliest stages of AD, when the first non-cognitive deficits appear, neuronal networks seem hyperactive [93–95]. This hyperactive state can cause epileptiform activity, as observed in patients with AD [96] and animal models [65, 95]. It was reported that transgenic hAPPJ20 mice have decreased levels of a voltage-gated sodium channel subunit (Nav1.1) on PV interneurons, leading to reduced inhibitory synaptic activity [65]. The dysfunction of PV interneurons likely contributes to epileptiform discharges in these mice [65]. Improper inhibition is possibly one of the main factors in neuronal hyperexcitability in AD [69]. In the following sections, we discuss other PV interneuron abnormalities that have been reported in relation to AD. Next, we outline selective PV interneuron manipulation as a potential mechanism to restore the proper functioning of neuronal networks in AD.

*Morphological changes.* PV interneurons exhibit highly plastic properties, and early in the disease, they may compensate for pathological changes and keep the E/I balance. To do so, their morphological and functional features change. In a mouse model with APP mutation, it was shown that the PV interneurons are resilient to amyloid-β plaques and can compensate morphologically for the amyloid-β-induced hyperactivity of pyramidal cells [97]. While the density of PV cells remains unchanged, the number of their terminals near plaques increases, and the synaptic area around initial segments significantly enlarges [97]. The increased dendrite length of PV interneurons and greater dendritic complexity were also described in 9- to 12-month-old APP/PS1 rats [98]. Similarly, enhanced synaptic projections of PV interneurons were observed in the hippocampus of 3-month-old APP/PS1 mice [61]. Together with morphological changes in the PV interneurons, higher levels of PV protein and GABAergic neuron marker (GAD67) were detectable in studied rats and mice, respectively [61, 98].

*Functional changes.* Functional changes may accompany morphological adaptations. Recently, it was proposed that the aberrant activity of PV interneurons changes throughout the disease, starting with hyperactive interneurons [63]. The authors showed the initial hyperexcitability of PV interneurons and gradual pathological changes in 4-month-old mice lead to aberrant synaptic transmission— reduction in excitatory input and inhibitory output— and their hypoactivation later in life, at about six months of age. Increased GABAergic inhibition might, in fact, act as a compensatory mechanism for overexciting pyramidal cells, which is caused by surrounding amyloid-β plaques [94, 95]. PV interneurons are more resilient to AD pathology [98, 99], and their activity is unaffected by the proximity of amyloid-β plaques [100].

Despite initial compensatory mechanisms, the PV interneurons are highly vulnerable to metabolic and oxidative stress during aging [101]. Further progression of AD takes its toll, and the PV interneurons start to malfunction, as indicated in the study of Hijazi et al. [63]. Decreased inhibitory transmission may then be the culprit in disrupted E/I balance and contribute to the progression of the disease, including brain hyperexcitability [70]. Indeed, decreased activity of PV interneurons was described in 8- to 11-month-old APP/PS1 mice [100]. Here, it is important to note that some interneurons might be affected by AD pathology even sooner than PV interneurons, for instance, somatostatin interneurons [102]. According to AD pathology, they increase their activity with proximity to the amyloid-β plaques and can cause or contribute to further hypoactivity of PV interneurons [94, 100]. However, their overall role in early AD and social cognition remains uncertain.

**Fig. 1:**
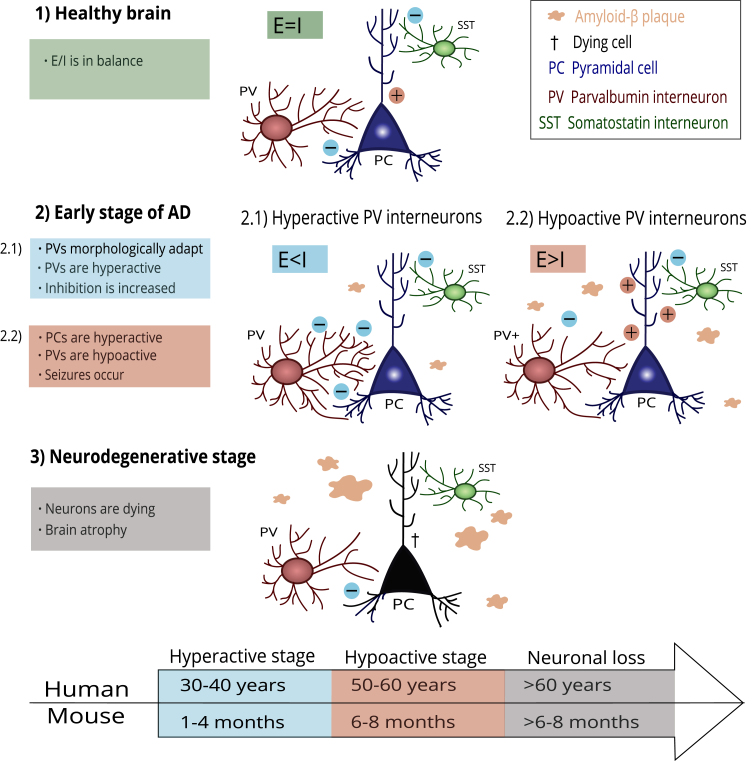
A possible mechanism of PV interneuron damage progression in AD. Healthy brains keep the E/I balance at a steady state (1). In the early stage of AD, the first amyloid-β plaques occur. PV interneurons adapt to this process through morphological and functional changes (2.1). PV interneurons become hyperactive, and the E/I balance is disrupted due to increased inhibition. However, PV interneurons cannot compensate for the damage and start hypofunction (2.2). Pyramidal cells increase firing due to decreased inhibition, and epileptiform seizures appear. In the final stage of AD, progressing pathological processes lead to irreversible damage and neuronal loss. The timeline under the scheme shows an illustrative comparison of AD-related histopathological changes in humans and mice according to their lifespan.

The impaired function of PV interneurons in hippocampal CA2 is accompanied by reduced inhibitory control in CA2, resulting in hyperexcitability of the pyramidal neurons and social memory deficits in Tg2576 mice [27]. This finding follows the general view that poor inhibitory transmission results in hyperexcitability of pyramidal cells, further leading to neurotoxicity and neuronal loss [103]. The PV interneuron damage progression in AD is illustrated in Fig. 1.

In contrast to previously reported hyperactivity in the hippocampus at approximately four months of age [63], other studies showed either hypoactivity or a reduced number of PV interneurons earlier in life. Reduced excitability of PV interneurons was observed in hAPPJ20 mice [72], similar to decreasing numbers in TgCRND8 mice [104] in already 1-month-old individuals. In addition, hypoactivity of PV interneurons was reported in the prefrontal cortex of APP/PS1 mice as early as nine to eleven weeks [105]. This inconsistency might arise from different animal models used and brain structures being studied, as summarized in Table 1. The variability in results could be also attributed to the particular research methods selected, e.g., *ex vivo* recording, which clearly differs from freely moving animals.

**Table 1:** Reported differences in PV interneuron morphology, excitability, and overall numbers

Study	Strain	Age	PV interneurons	Structure
Mahar et al. (2017) [104]	TgCRND8 mice	1 m	↓ number	hippocampus
Mondragón-Rodríguez et al. (2018) [72]	hAPPJ20 mice	1 m	↓ excitability	subiculum
Shu et al. (2023) [105]	APP/PS1 mice	2-3 m	↓ activity	prefrontal cortex
			↓ number
Hollnagel et al. (2019) [61]	APP/PS1 mice	3 m	↑ density of projections	hippocampus
			↑ PV immunoreactivity
Hijazi et al. (2020) [63]	APP/PS1 mice	4 m	↑ excitability	hippocampus
		6 m	↑ PV immunoreactivity
			no difference in number
			↓ activity
			↓ PV immunoreactivity
			no difference in number
Algamal et al. (2022) [100]	APP/PS1 mice	8–11 m	↓ activity	somatosensory cortex
Verret et al. (2012) [65]	hAPPJ20 mice	4–7 m	↓ Nav1.1 subunit levels	parietal cortex
			↓ amplitude of AP
Li et al. (2022) [107]	5xFAD mice	5-6 m	↓ PV levels	hippocampus
			↓ number
Ali et al. (2020) [103]	5xFAD mice	6–9 m	↓ number	frontal cortex
Rey et al. (2022) [27]	Tg2576 mice	9 m	↓ number	hippocampus
Giesers & Wirths (2020) [108]	5xFAD mice	12 m	↓ number	hippocampus
	Tg4-42 mice	12 m	no difference in number
Mackenzie-Gray Scott et al.	5xFAD mice	3–6 m	no difference in number	hippocampus
(2022) [99]		12+ m	↓ number
Takahashi et al. (2020) [111]	APP/PS1 mice	10 m	↓ number	hippocampus
Sun et al. (2021) [110]	TgF344-AD rats	9 m	↓ PV levels	hippocampus
Morrone et al. (2022) [98]	TgF344-AD rats	9–12 m	↑ complexity and/or length of dendrites	hippocampus
		15 m	↓ complexity and/or length of dendrites
Sos et al. (2020) [97]	APP^NL - F^ mice	18 m	↑ enlarged synapses	hippocampus
			no difference in density
Zallo et al. (2018) [109]	3xTg-AD mice	18 m	↓ number	hippocampus

It should be pointed out that PV interneuron dysfunction might be place- and cell-type specific, which is supported by the observation that the basket cells, but not the bistratified or axo-axonic cells, had reduced activity during memory consolidation in the hippocampal CA1 region of 3-month-old 5xFAD mice [62]. To our knowledge, no study has addressed the impairment of specific cell types in dorsal CA2, where social memories form.

### PV interneurons in the neurodegenerative stage

Studies across human and animal research have reported a decline in PV interneuron numbers. Immunohistochemical analysis of the brains of 70–80 years old AD patients showed over 60% loss of PV interneurons in some hippocampal subfields (dentate gyrus, CA1, and CA2), compared to healthy brains [106]. Similarly, loss of PV or PV interneurons was described in the hippocampus of various AD models with amyloid-β plaque accumulation [107–110]. Takahashi et al. reported a 50% reduction of PV interneurons in the hippocampus of 10-month-old APP/PS1 mice [111], similar to the loss of pyramidal cells reported in APP^SL^PS1KI mice of the same age [112]. Most research studies use anti-PV antibodies as a marker for counting PV interneurons. However, the decrease in staining when using anti-PV antibodies does not necessarily account for neuronal loss; decreased number of PV interneurons can also be explained by decreased PV expression in the neuronal cells [113]. It is crucial to differentiate between these two phenomena as they may have opposing effects. Some studies suggest that PV cell loss leads to decreased inhibition, whereas loss of PV in cells slows the decay of Ca^2 +^ kinetics and enhances synaptic facilitation, which in turn favors inhibition [113–116]. It is, therefore, questionable if the observed decrease in PV interneurons reflects the actual neuronal loss. The number of PV interneurons was reported to be reduced as early as in 1-month-old TgCRND8 mice [104] and 2-to 3-month-old APP/PS1 mice [105]. Thus, the early PV reduction delineated in these studies might reflect early deficits in PV expression rather than dying PV interneurons.

### Manipulating the PV interneuron activity

One advantage of animal models is the possibility of interrogating the function of interneurons and seeing their role in AD-related behavior. For example, enhancing GABAergic activity directly by GABA administration was already shown to improve cognitive performance in 2-month-old APP/PS1 mice [117]. Targeted manipulations of selected cells or brain circuits with the potential to modulate the E/I balance may further help to expand the knowledge about altered neuronal functioning or alleviate cognitive decline. Hijazi et al. showed that chemogenetic inhibition of PV interneurons earlier or activation later in life led to similar improvements in cognitive functioning and proposed opposite interventions according to the specific stage of the disease [63]. Furthermore, chronic optogenetic stimulation (40 Hz) of PV interneurons in the medial septal region of mice with double APP mutation restored the impaired slow gamma (30–60 Hz) oscillations in the hippocampus and supported memory retrieval [64]. Non-invasive 40 Hz visual stimulation was as efficient as the modulation of hippocampal activity and improved spatial recognition memory of the 5xFAD model of AD [118]. Such visual stimulation also lowered amyloid-β levels in the visual cortex [119]. It would be interesting to test how such stimulation affects social behaviors or recognition in early AD animal models. In another approach, we can target social modulators to alleviate early symptoms in the social domain. To achieve this, we consider OT a promising drug and discuss its benefits in the following section.

## OXYTOCIN AND SOCIAL COGNITION

OT is a neuropeptide involved in many aspects of social behaviors, including empathy or social recognition [120–122]. Here, we will put oxytocinergic signalization in the context of social memory processing and focus on their modulatory outcomes in PV interneurons. As social memory is crucially dependent on dorsal hippocampal CA2 [91, 123, 124], we will highlight the modulation of this part.

Modulatory effects of OT in the hippocampus were already comprehensively described in another review [125]. In summary, the main oxytocinergic pathway leading to the hippocampus originates in the paraventricular nucleus of the hypothalamus [126]. OT acts on neurons at both presynaptic and postsynaptic levels [127], exerting widespread effects via G-protein coupled receptors [128]. Most notably, OT enhances the long-term potentiation in the hippocampal CA1 by activating protein kinase M*ζ* [129]. These processes are essential for maintaining long-term memories [130].

The hippocampal CA2/CA3 regions in mice are highly enriched in OTRs, and about 90% of hippocampal OTRs are expressed on excitatory pyramidal cells [91, 123]. Despite the relatively low presence of OTRs in GABAergic interneurons, about 50% of PV interneurons in the dorsal CA2/CA3 regions express OTRs [90, 91]. The high abundance of OTRs in CA2 led scientists to an assumption that these receptors might play a role in social memory. Indeed, deleting these receptors in the hippocampus led to impairments in discrimination between a novel and familiar mouse [91] and negatively affected the long-term storage of social memories, without affecting sociability per se [92, 131]. Moreover, oxytocinergic signalization is necessary for long-term social memory formation and persistence [131]. It is important to note that OT shapes neural activity in the hippocampus and other brain areas related to social behavior and memory, especially the amygdala and prefrontal cortex [132].

In general, OT increases the excitability of GABAergic interneurons, favoring inhibition [133]. This interneuronal depolarization is cell-specific and restricted to PV interneurons [134]. Therefore, OT indirectly inhibits the activity of pyramidal cells in hippocampal CA1 [135]. OTRs can similarly be found in the ventral part of CA1 [91]; however, to this date, no study has probed the effects of OT administration in this area on social recognition. Using a selective OTR agonist TGOT in the hippocampal CA2, it was found that PV interneurons and excitatory pyramidal cells are activated together [90]. The authors proposed that co-activation is necessary for shaping the burst-firing of pyramidal cells, causing a rapid and precise sequence of action potentials and modulating synaptic plasticity [90, 134]. Nevertheless, the exact mechanisms of the E/I balance modulation induced by OT in complex neural networks lie upon further investigation. Considering the profound effect on PV interneurons, the OT administration may be beneficial for improving some aspects of social cognition, including memory. This modulation might have more significant effects at certain stages of the disease, e.g., when the interneurons are hypoactive.

### Oxytocin as a potent future modulator in AD

The therapeutic effects of OT have already been probed in other neuropsychiatric disorders, such as anxiety disorders, depression, and schizophrenia [136]. While autism is characterized by disruption in oxytocinergic signalization [137], AD patients seem to have unaltered OT plasma concentrations [138] and the number of OT-expressing neurons [139]. Therefore, administering OT in AD could function as a positive modulator of brain functions rather than a correction for impaired signaling. According to the ClinicalTrials.gov database, one clinical testing of intranasal OT administration for frontotemporal dementia is underway (NCT03260920), with three others done in the past (NCT01386333, NCT01937013, NCT01002300). In contrast, the effects of OT have not been tested yet in patients with AD and its influence on social memory is still limited to research on rodent AD models.

OT was found to improve social memory after a 2-h re-exposure interval when injected into the septal nuclei of rats [140]. Later studies revealed that OT in the hippocampus and medial amygdala has a positive impact on social memory [91, 141]. The OT levels rise significantly in the amygdala and hippocampus in mice 30 min after intranasal administration, proving its effects on the brain [142].

An increasing body of evidence suggests the beneficial effects of OT in the AD-affected brain. Recent findings reveal that OT can improve long-term potentiation in the hippocampus that is impaired due to the amyloid-β plaques, thereby facilitating the transmission of information between synapses [143]. In a further study, the authors injected OT in the ventricles or administered an OT derivative intranasally in mice with amyloid-β peptide-induced amnesia and observed memory improvements in spatial domains [144]. Neuroprotective effects of OT were described when using an aluminum-induced AD model in female rats [145]. The authors stated that OT administered daily for eight weeks suppressed pathological pathways associated with amyloid-β accumulation, reduced tau levels, acetylcholinesterase activity, and decreased levels of kinases promoting neuronal death, such as ERK1/2. Another study confirmed in APP/PS1 mice that a 6-week prolonged intranasal administration of OT reduced amyloid-β plaque size in the hippocampi and promoted the deposition in dense core plaques, which is thought to be a neuroprotective mechanism [146]. OT also has anti-inflammatory properties and can attenuate microglial overactivation, which is typical for the disease [146]. Moreover, OT administration rescued the social memory deficits in these mice [146]. Activation of OTRs also promotes hippocampal neurogenesis [147], which further augments the potential positive effects of OT treatment.

**Fig. 2:**
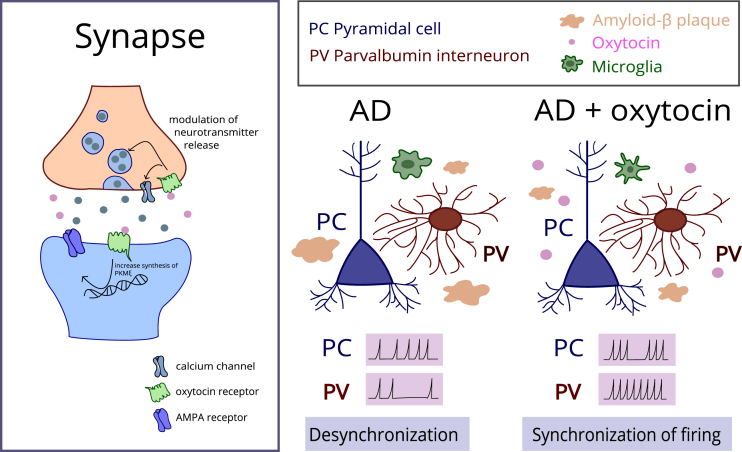
A schematic illustration of oxytocin effects on synapse and its potential therapeutic effects in AD brains. Oxytocin induces long-term potentiation by increasing levels of protein kinase M*ζ*, which regulates transcription of the AMPA receptors and their incorporation into the membrane [129, 148]. Oxytocin treatment may help to rescue the E/I imbalance in AD-affected brains, synchronize the firing of pyramidal cells by modulating the activity of PV interneurons, reduce microglial overactivation and support neuroprotective mechanisms.

As outlined in the previous chapter, the precise mechanisms of OT on E/I balance are yet to be fully elucidated. Since OT promotes the activity of PV interneurons, its administration might strengthen the inhibitory tone and enhance burst firing, which could facilitate synaptic transmission between hippocampal CA2 and CA1 regions, e.g., during consolidation of social memories [90]. In addition, enhanced inhibitory activity might reduce epileptiform activity of neuronal networks [65]. The OT effects on the synapse and possible mitigation of some aspects of AD pathology are illustrated in Fig. 2.

Considering some limitations and discrepancies regarding oxytocinergic signalization in humans is essential. Rodent neural structures, like the hippocampus, may differ across species. For example, guinea pigs lack OTRs in the hippocampus [149]. The incomplete understanding of oxytocinergic signaling in humans poses potential challenges in translational research. However, it also presents an entirely new avenue for investigation that awaits exploration.

## CONCLUDING REMARKS

Abnormal activity in GABAergic systems and observed altered E/I balance in humans with AD and its animal models show apparent involvement of PV interneurons. A growing interest has emerged in investigating their role during the early stages, preceding the full manifestation of the disease. Particular attention has been directed toward early changes in social cognition and social memory, aiming to establish a connection between early deficits and the aberrant activity of the PV interneurons. The relation between PV interneurons activity and social behavior in AD might be found more complex if proven true that PV interneurons transit from a hyperactive to a hypoactive state during the progression of the disease.

OT is likely a potent modulator of PV interneurons and social cognition, suggesting a prospective role in treating cognitive and social deficits. Although some clinical trials already included intranasal OT administration to alleviate some neuropsychiatric conditions, the focus on therapeutic effects in AD is still minor. Moreover, precise mechanisms of action and modulatory effects of OT need to be clarified in future studies, with the inevitable role of animal models. It would be interesting to test the OT’s local effects in socially engaged brain structures, such as the hippocampus, amygdala, or prefrontal cortex, and distinguish its modulatory role in specific cell types. Alternatively, the effects of OT may vary based on its administration at different phases, for example, during encoding, consolidation, or recall of social memories.
